# Sparing the hippocampus and the hypothalamic- pituitary region during whole brain radiotherapy: a volumetric modulated arc therapy planning study

**DOI:** 10.1186/s12885-020-07091-x

**Published:** 2020-06-30

**Authors:** P. Mehta, S. Janssen, F. B. Fahlbusch, S. M. Schmid, J. Gebauer, F. Cremers, C. Ziemann, M. Tartz, D. Rades

**Affiliations:** 1 Department of Radiation Oncology, University of Lübeck, Lübeck, Germany; 2 Private Practice of Radiation Oncology, Hannover, Germany; 3 Department of Pediatrics and Adolescent Medicine, Friedrich-Alexander-University of Erlangen-Nürnberg, Erlangen, Germany; 4 Institute for Endocrinology and Diabetes, University of Lübeck, Lübeck, Germany; 5 German Center for Diabetes Research (DZD), Neuherberg, Germany

**Keywords:** Whole brain radiotherapy (WBRT), Brain metastases, Hippocampus sparing, Hypothalamus, Pituitary gland, Volumetric modulated arc therapy (VMAT)

## Abstract

**Background:**

Feasibility testing of a simultaneous sparing approach of hippocampus, hypothalamus and pituitary gland in patients undergoing whole-brain radiotherapy (WBRT) with and without a concomitant boost to metastatic sites.

**Introduction:**

Cognitive impairment and hormonal dysfunction are common side effects of cranial radiotherapy. A reduced dose application to the patho-physiologically involved functional brain areas, i.e. hippocampus, hypothalamus and pituitary gland, could reduce these common side effects. While hippocampal sparing is already a common practice to improve cognitive outcome, technical experience of additional combined sparing of the hypothalamus/pituitary gland (HT-P) is insufficient.

**Methods:**

Twenty patients were included in the planning study. In 11 patients, a total dose of 36 Gy of WBRT (2 Gy per fraction) plus a simultaneous integrated boost (SIB) of 9 Gy (0.5 Gy per fraction, total dose: 45 Gy) to the brain metastases was applied. In 9 patients, prophylactic cranial irradiation (PCI) was simulated with a total dose of 30 Gy (2 Gy per fraction). In both patient cohorts, a sparing approach of the hippocampus and the HT-P area was simulated during WBRT. For all treatment plans, volumetric modulated arc therapy (VMAT) was used. Quality assurance included assessment of homogeneity, conformality and target coverage.

**Results:**

The mean dose to the hippocampus and HT-P region was limited to less than 50% of the prescribed dose to the planning target volume (PTV) in all treatment plans. Dose homogeneity (HI) of the target volume was satisfying (median HI = 0.16 for WBRT+SIB and 0.1 for PCI) and target coverage (conformation number, CN) was not compromised (median CN = 0.82 for SIB and 0.86 for PCI).

**Conclusion:**

Simultaneous dose reduction to the hippocampus and the HT-P area did not compromise the PTV coverage in patients undergoing WBRT+SIB or PCI using VMAT. While the feasibility of the presented approach is promising, prospective neurologic, endocrine outcome and safety studies are required.

## Background

Up to 30% of cancer patients develop brain metastases during their disease [1]. Despite the increasing use of high precision radiation techniques for small volumes [2–4], whole brain radiotherapy (WBRT) remains the treatment of choice for patients with multiple brain metastases [5] as well as for prophylactic cranial irradiation (PCI) in patients with small cell lung cancer (SCLC) [6, 7]. However, cognitive and neuroendocrine impairment following cranial radiotherapy remains a concern. Mechanistically, damage to the stem cells within the hippocampus might play a major role in the observed memory decline [8]. In line with this finding, Gondi et al. were able to show that conformal avoidance of the hippocampus during WBRT was associated with preservation of memory function and quality of life (QoL), as compared to a non-sparing historical series [9]. Apart from neurocognitive decline, another common sequela of cranial radiation therapy is functional endocrine impairment due to critical doses to the hypothalamus and the pituitary gland. A significant percentage of patients with brain tumors [10–12] and head and neck cancer [13–15] develop hormonal deficiencies after radiotherapy. At the time of our research period, endocrine follow-up data after WBRT was scarce. Nevertheless, it has been shown that hormonal changes can occur after applied doses as low as 18 Gy in patients with radiotherapy to head and neck cancers and brain tumors [13]. This dichotomy led us to investigate a combined sparing approach involving both the hippocampal and the hypothalamus/pituitary gland (HT-P) area during WBRT. In our planning study we examined the feasibility of such an approach using volumetric modulated arc therapy (VMAT).

## Methods

**Fig. 1:**
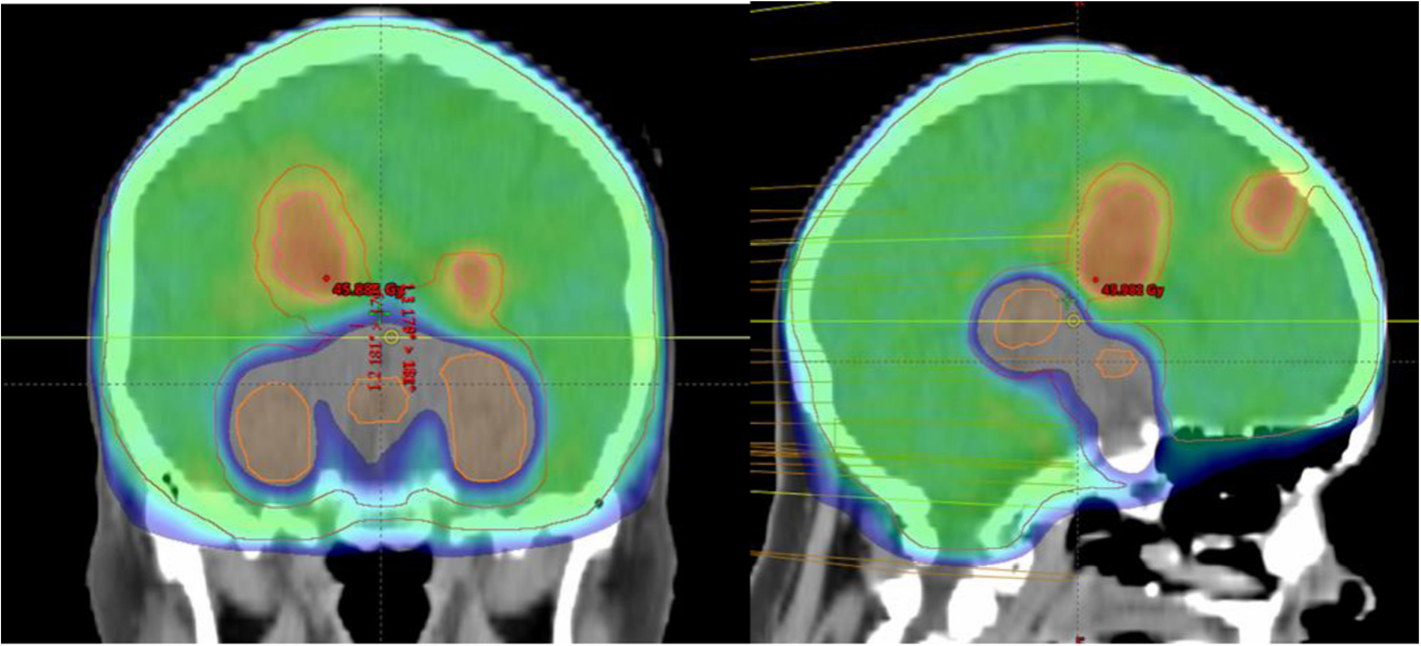
a and b Representative example of a VMAT plan with avoidance of the hippocampus and HT-P area and SIB (color-wash, coronar (**a**) and sagittal (**b**) view, dose levels: blue: 25–32 Gy, cyan: 32–37 Gy, green: 37–42 Gy, yellow: 42–44 Gy, orange: 44–46 Gy, red: > 46 Gy)

The computed tomography (CT)-data sets of 20 patients who previously received WBRT in our institution from 2017 to 2019 were included. The CT-scans were performed with a Siemens Biograph 40 m with a slice thickness of 3 mm. To facilitate contouring of the brain structures and metastases T1-weighted contrast-enhanced magnetic resonance images (MRI) were fused to the planning CT. In addition to the contoured hippocampus (according the RTOG 0933 study [9]), the hypothalamus and pituitary gland (including the pituitary stalk) were contoured and planning risk volumes (PRV) were created using a 5-mm margin [16, 17]. CT data sets with metastases within 5 mm around the avoidance structures were excluded from this planning study. Further, the whole brain planning target volume (PTV) was contoured and cropped by the hippocampus and HT-P as a planning risk volume (PRV). An auxiliary PTV structure consisting of the part of the optimization PTV surrounding the HT-P and hippocampus helped to control the dose drop in the immediate vicinity of the hippocampus and the HT-P (Fig. 1).

Treatment plans were then created using Eclipse 15.5 (Varian Medical Systems, Inc., Palo Alto, CA, USA) for a Clinac DHX linear accelerator equipped with a Millennium 120 MLC. The Photon Optimizer (PO) and Anisotropic Analytical Algorithm (AAA), both in versions 15.5.11, were utilized [18]. The normalization point was set to 100% of the mean dose of the target volume. All treatment plans were created on the basis of sparing both the hippocampal and the HT-P area, concomitantly avoiding dose peaks to lenses, eyes, chiasm, optical nerves and brainstem. 11 of 20 patients were planned with a dose of 18 × 2 Gy = 36 Gy as a WBRT with a SIB (18 × 0.5 Gy = 9 Gy, total dose: 45 Gy) to the metastases resulting in a total dose of 18 × 2.5 Gy in the area of the SIB, whereas the other 9 patients were planned as a PCI with a dose of 15 × 2 Gy = 30 Gy. A VMAT treatment plan was generated individually for each patient by a medical physicist (P.M.). Table 1 summarizes patient and treatment parameters.

**Table 1:** Patient and treatment related parameters, HI = homogeneity index, HT-P = hypothalamus-pituitary area, CN = conformation number, SIB: simultaneous integrated boost, n.a. =not applicable; m = male, f = female

	PTV volume [cm^3^]	Hippocampal volume [cm^3^]	Hippocampal HT-P- + 5 mm volumes V_PRV_ [cm^3^]	Number of metastases	Metastases volumes [cm^3^]	% SIB of PTV	D95 (%)	Hippocampal HT-P- + 5 mm mean dose D_PRV_ [Gy]	HI	CN
1	1748.1	12.6	58.9	n.a.	n.a.		96.4	12.8	0.09	0.85
2	2048.5	12.6	58.0	n.a.	n.a.		95.9	14.1	0.17	0.86
3	2140.7	6.1	43.9	n.a.	n.a.		95.5	14.6	0.12	0.86
4	2083.5	10.7	65.6	n.a.	n.a.		96.4	14.2	0.09	0.86
5	1974.4	4.5	43.2	n.a.	n.a.		95.3	14.9	0.12	0.86
6	1797.2	4.7	59.6	n.a.	n.a.		95.3	14.9	0.11	0.84
7	1805.3	8.1	51.4	n.a.	n.a.		96.7	15	0.08	0.86
8	1521.4	10.4	58.8	n.a.	n.a.		96.4	14.9	0.09	0.82
9	1991.4	6.7	45.9	n.a.	n.a.		96.4	15	0.09	0.87
10	1593.9	7.4	47.9	2	5.2	0.33	98.1	15.1	0.11	0.84
11	1924.7	22.4	51.2	1	9.4	0.49	98.1	15	0.13	0.85
12	1761.8	2.7	38.9	2	7.1	0.40	98.0	14.1	0.14	0.84
13	1436.4	4.0	39.6	1	15.9	1.11	97.8	14.9	0.19	0.81
14	1860.0	4.7	44.1	1	4.1	0.22	98.0	13.2	0.16	0.86
15	1802.7	6.0	42.2	9	31.7	1.76	97.6	14	0.214	0.79
16	2286.7	10.1	65.8	3	73.0	3.19	97.3	14.2	0.16	0.79
17	1641.1	8.8	53.6	1	6.5	0.40	98.3	13.6	0.17	0.85
18	2029.0	9.6	59.4	1	105.0	5.17	97.5	15.4	0.15	0.72
19	1941.3	2.5	34.3	4	20.6	1.06	97.6	14.8	0.23	0.82
20	1781.4	5.8	41.5	2	19.8	1.11	97.6	15	0.13	0.82

Two different planning approaches were used for therapeutic WBRT with SIB to metastases and for PCI. Both were planned with three 6 Megavolt (MV) photon beam full arcs using the VMAT technique. For the treatment plans including SIB two of the arcs had a rotation collimator of 320° and 40°, while in the third arc the collimator was rotated by 90° and the jaws were adjusted to the length of the organs of risk (OAR), i.e. the hypothalamus, hippocampus and pituitary gland to guarantee homogenous dose coverage between the OAR. For the PCI plans the collimator angles were 280°, 90° and 11° respectively. Couch rotations of 15°, 0°, and 345° were used. These different techniques provided the optimal combined sparing approaches of both hippocampal/HT-P structures and ocular lenses, while concomitantly ensuring the best PTV-dose coverage.

The treatment plans were generated with the goal to achieve a dose of lower than 50% of the prescription dose of the PTV in the sparing regions without compromising conformal dose coverage. Additionally, mean maximum doses to the ocular lenses were kept below 10 Gy. Homogeneity index (HI) was calculated as follows [19]:

$$ \mathrm{HI}=\frac{{\mathrm{D}}_{2\%}\hbox{-} {\mathrm{D}}_{98\%}}{{\mathrm{D}}_{\mathrm{median}}} $$

Where D_x%_ is the dose which is at least delivered to x% of the volume and D_median_ is the median dose. Smaller values of HI correspond to a more homogeneous irradiation of the target volume, and a value of 0 corresponds to a completely homogeneous dose distribution within the target [16].

Conformality index or conformation number (CN) was calculated according the formula introduced by van’t Riet et al. in 1997 [20]:

$$ \mathrm{CN}=\frac{{\mathrm{TV}}_{\mathrm{RI}}}{\mathrm{TV}}\times \frac{{\mathrm{TV}}_{\mathrm{RI}}}{{\mathrm{V}}_{\mathrm{RI}}} $$

Where TV_RI_ is target volume covered by the reference isodose (95% isodose), TV is the target volume and V_RI_ is the volume of the reference isodose (95% isodose). The conformation number reaches a value between 0 and 1. A value of 1 represents a reference isodose covering the exact target volume without irradiation of healthy tissue and indicates optimal conformation. On the other hand, a value of 0 equals no conformation at all [20].

The target coverage (TC) was measured as the volume within the target receiving a dose greater or equal to the prescription dose (VTpres) divided by the target volume (TV) [21].

$$ \mathrm{TC}=\frac{{\mathrm{V}}_{\mathrm{Tpres}}}{\mathrm{TV}} $$

No patients consent was obtained as all patients’ data were irreversibly anonymized before analysis. The in silico analysis included CT database data only. In this form, the study was approved by the local ethics committee of the University of Lübeck, Germany (reference number: 19-075A).

## Results

**Fig. 2:**
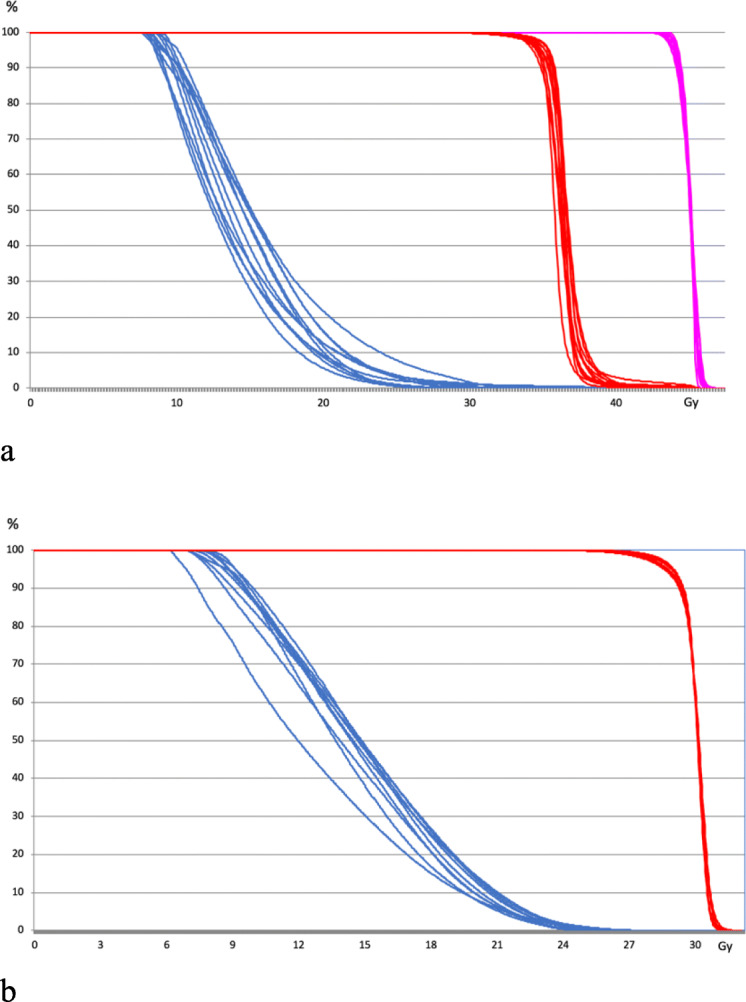
a and b Representative cumulative dose volume histograms (DVH) for SIB plans (**a**) and PCI plans (**b**). Blue: sparing region, red: PTV whole brain, pink: SIB

The median total brain volume including the avoidance region was 1832.7 cm^3^ (range: 1436.4 cm^3^–2286.7 cm^3^). The median volume of the hippocampus/HT-P area (including a margin of 5 mm) was 43.9 cm^3^ (range: 34.3–65.8 cm^3^). For patients receiving a SIB to brain metastases, the median value of the SIB volume was 15.9 cm^3^ (range: 5.2–105.0 cm^3^). Number of metastases treated with a SIB ranged from 1 to 9 (median: 2). The median percentage of the SIB volumes of the entire planning treatment volume (PTV) was 3.3% (range: 1.3–11.6%). In the 11 WBRT plans including SIB to brain metastases the median delivered dose to the hippocampus/HT-P area was 14.9 Gy (range: 13.2–16.2 Gy). In the 9 PCI plans the delivered dose to the hippocampus/HT-P could be held below 15Gy (median: 14.8 Gy, range: 12.8–15.0 Gy). Maximum dose to the ocular lenses was limited to 10 Gy for each patient. Median maximum dose for all plans within in lenses was 8.3 Gy. The corresponding values for the eyes, the brainstem, chiasma and optic nerves were 25/30 Gy, 31/40 Gy, 29/33 Gy and 31/39 Gy for prophylactic and therapeutic plans, respectively. The median homogeneity index was 0.16 (range: 0.11–0.23) for the SIB plans and 0.10 (range: 0.08–0.17) for the PCI plans. The median D_95%_ for the WBRT plans including SIB 97.8% (range: 97.3–98.3%) and 96.4% (range: 95.5–96.7%) for PCI plans. The median conformity index was 0.85 for all plans, 0.82 for the therapeutic plans including SIB (range: 0.72–0.86) and 0.86 for the PCI plans (range: 0.82–0.87). The target coverage was 0.7 (range: 6.3–8.7) for prophylactic and therapeutic plans, respectively. Figure 2a and b show the dose volume histograms (DVH) for SIB plans and PCI plans.

## Discussion

WBRT for brain metastases can impair neuro-cognitive functions in terms of memory loss and reduced QoL [8]. Neural stem cells within the hippocampus may play an important role in this patho-mechanism. In RTOG 0933, avoidance of the hippocampus during WBRT was associated with preservation of memory and QoL as compared with a non-sparing historical series [9]. Preliminary analysis of a randomized phase III trial confirms the hypothesis of preserved neurocognitive function while achieving similar intracranial control and survival [22].

Functional endocrine deficiencies after brain radiotherapy are common [23]. Long term follow-up studies indicate that radiation induced HT-P dysfunction may occur in up to 80% of patients and is often associated with an adverse impact on growth, body image, skeletal health, fertility, sexual function and physical and psychological health [24]. Several studies showed the hormonal impairment to be dose-dependent with an increased incidence at doses above 30 Gy [17]. Until now, most data of radiation induced endocrine sequelae in adults originate from patients being treated for head and neck cancer and non-pituitary brain tumors. Endocrine follow-up data on hormonal changes after WBRT are scarce [23]. As the hormonal impairment is described to be dose-dependent, limiting the dose to the HT-P area could be beneficial. During WBRT, this could be realized with a sparing approach analogue to the hippocampus sparing technique introduced by Gondi et al. [9], as previously discussed by us [23]. Arguments against a theoretical benefit of such a sparing approach are the limited life expectancy of patients with brain metastases and lower doses to the HT-P region compared to RT in head and neck cancers and brain tumors. Still, in a current review of literature we could reveal a potential effect of RT for doses of less than 30 Gy being within the dose range of WBRT [23]. Moreover, the potential negative endocrine effect might already occur as early as within the first year after RT [23]. This is of relevance especially for patients with a more favorable prognosis, e.g. for patients with good performance status and a limited tumor burden or in the prophylactic setting in SCLC patients. For this reason, we carried out a planning approach for both, therapeutic and prophylactic scenarios encompassing a combined sparing of the hippocampus and the HT-P area.

According to the present VMAT planning study, simultaneous sparing the hippocampus and the HT-P axis was feasible. The dose to the avoidance regions could be limited to less than 50% of the prescribed doses to the PTV. For the hippocampus, several dose constraints were suggested in previous studies. In the RTOG 0933 protocol, the dose to 100% of hippocampus did not exceed 9 Gy (D_100%_ < 9 Gy), and the maximal hippocampal dose did not exceed 16 Gy [9]. Other studies involving hippocampal sparing approaches in patients treated with WBRT delivered mean doses to the hippocampi ranging from 5 Gy to 20 Gy, depending on radiation techniques and total doses [24]. Until now, no threshold dose for the HT-P area has been established. Kyriakakis et al. assessed the effects of cranial RT on pituitary function in adults with gliomas distant to the HT-P axis. The dose exposure of the HT-P axis was correlated with individual axis dysfunction to establish dose thresholds. The authors argued for the implementation of long-term endocrine surveillance in RT cases exceeding 30 Gy to the HT-P axis [25].

In a study by Fan et al., in which the hippocampus and the HT-P area were spared simultaneously using intensity modulated radiotherapy (IMRT), the hippocampus received a mean dose of 9.6 Gy, and the hypothalamus and the pituitary gland mean doses of 11.06 and 10.66 Gy, respectively [16]. In the present study, the mean doses to the hippocampus and the HT-P area were 15 Gy i.e. comparably higher. This finding might result from higher doses to the total brain volume in our study when compared to previous studies (36 Gy and 30 Gy versus 30 Gy and 25 Gy) [9, 16]. In the present study, the metastases even received 45 Gy. Moreover, we also attempted to spare the ocular lenses during WBRT and to achieve conformal dose coverage. However, our boost doses to a maximum 45Gy (normo-fractionated) are a rather cautious approach and are currently under discussion.

In contrast to Fan et al., who were the first group describing a combined sparing approach of the hippocampus and the HT-P area using IMRT, we chose a VMAT approach. For hippocampal sparing during WBRT (without the HT-P area), the use of VMAT was shown to significantly improve dose distribution in terms of target coverage and homogeneity [26–28]. In the study of Sood et al., the use of a VMAT-technique also reduced mean and maximum doses to other organs at risk (OAR) such as cochleae and parotid glands [29]. These promising results inspired us to use VMAT in our approach to spare the hippocampus and the HT-P. In addition, when comparing our VMAT data to the IMRT approach used by Fan et al., we were able to achieve less heterogeneity with respect to the dose coverage of the PTV (homogeneity index: 0.23 vs. 0.10 and 0.16 in our study). Further, we kept the maximum dose to the ocular lenses below 10 Gy; no information concerning the doses to the lenses was provided by Fan et al. [16]. Another advantage of VMAT is its faster treatment delivery. For hippocampal sparing in WBRT, Wang et al. demonstrated a significant shorter treatment time of approximately 25% using VMAT in comparison to IMRT [30]. Moreover, Rong et al. found faster treatment delivery of VMAT when compared to IMRT [31].

For hippocampal sparing in WBRT, slightly superior homogeneity indices and target coverage were found for tomotherapy when compared to IMRT and VMAT [31, 32]. However, the availability of tomotherapy is limited, and the treatment planning time is significantly longer. Another possibility to improve the quality of the treatment planning could be an inclined head position [33, 34]. In a recently published study, Zheng et al. showed feasibility using VMAT and tomotherapy for HT-P and hippocampal axis sparing for cranio-spinal irradiation. They also found that VMAT was able to achieve good conformality [35].

In line with data from hippocampal sparing WBRT, simultaneous sparing of the hippocampus and HT-P via VMAT delivered highly conformal and fast-to-apply treatment plans, resulting in a direct advantage for patients in their daily treatment sessions.

A sparing approach of certain brain regions bears the risk of jeopardizing oncologic outcomes in terms of intracranial control and consecutive overall survival. Therefore, the estimated risk of metastases within spared structures and their proximity have to be taken into careful consideration. Gondi et al. deemed hippocampus sparing WBRT safe with an estimated risk of peri-hippocampal metastases of 8.6% [36]. Our group has recently analyzed 865 patients with 4280 metastases and showed an incidence of involvement of the HT-P area of approximately 4% [37]. Against that background, an approach of sparing the HT-P area in addition to the hippocampus during WBRT appears reasonable. Thus, in order to reveal a clinical meaningful effect of HT-P region sparing within WBRT, a prospective study is planned evaluating a sparing approach with simultaneous avoidance of the hippocampus and the HT-P area including endocrine follow-up. The current planning study, which is a prerequisite for the planned prospective trial, showed technical feasibility of such an approach using VMAT even for dose escalation with a SIB. In the absence of safety data, the presented approach remains experimental and should not be applied outside a clinical study.

## Conclusion

Simultaneous dose reduction to the hippocampus and the HT-P area did not compromise the PTV coverage in patients undergoing WBRT+SIB or PCI when using VMAT. While the feasibility of the presented approach is promising, prospective neurologic and endocrine outcome studies are required to properly evaluate the usefulness of such an approach.

## Data Availability

The datasets used and/or analyzed during the current study are available from the corresponding author on reasonable request.

## Abbreviations

CN: Conformation number
DVH: Dose volume histograms
IMRT: Intensity modulated radiotherapy
HI: Homogeneity index
HT-P: Hypothalamus/pituitary gland
PCI: Prophylactic cranial irradiation
PTV: Planning target volume
SCLC: Small cell lung cancer
SIB: Simultaneous integrated boost
TC: Target coverage
OAR: Organs at risk
VMAT: Volumetric modulated arc therapy
WBRT: Whole brain radiotherapy
